# Do novo del(9)(p13) in a childhood T-cell prolymphocytic leukemia as sole abnormality

**DOI:** 10.1186/2162-3619-3-28

**Published:** 2014-11-25

**Authors:** Abdulsamad Wafa, Abdulmunim Aljapawe, Moneeb AK Othman, Thomas Liehr, Eyad Alhourani, Walid Al Achkar

**Affiliations:** Human Genetics Division, Department of Molecular Biology and Biotechnology, Atomic Energy Commission of Syria, P.O. Box 6091, Damascus, Syria; Molecular Biology and Biotechnology Department, Mammalians Biology Division, Flow-cytometry Laboratory, Atomic Energy Commission of Syria, P.O. Box 6091, Damascus, Syria; Jena University Hospital, Institute of Human Genetics, Kollegiengasse 10, Jena, D-07743 Germany

**Keywords:** Childhood T-cell prolymphocytic leukemia (T-PLL), Chromosomal aberrations, Fluorescence in situ hybridization (FISH), Multicolor banding (MCB)

## Abstract

T-cell prolymphocytic leukemia (T-PLL) is a rare and aggressive subtype of chronic lymphocytic leukemia. Usually it presents in older people with a median age of 61 years. T-PLL is characterized by elevated white blood cell (WBC) count with anemia and thrombocytopenia, hepatosplenomegaly, and lymphadenopathy; less common findings are skin infiltration and pleural effusions. The most frequent chromosomal abnormalities in T-PLL include 14q11.2, chromosome 8, and 11q rearrangements. Also deletions in the short arm of a chromosome 9 are reported in ~30% of T-PLL together with other aberrations. Here we report a childhood T-PLL case with a de novo del(9)(p13) as sole acquired anomaly leading to monosomy of the tumor suppressor gene *CDKN2A* (cyclin-dependent kinase inhibitor 2A). Also, to the best of our knowledge this is the first case of a childhood T-PLL with this aberration.

## Introduction

T-cell prolymphocytic leukemia (T-PLL) is an aggressive lymphoproliferative disorder that represents in 2% of all mature lymphocytic leukemias in adults; also it has been classified as an aggressive subtype of chronic lymphocytic leukemia (CLL) [1]. T-PLL causes major problems due to negative effects on the immune system, thus predisposing the affected patient to a variety of infections, and possibly death [2]. T-PLL affects mainly adults (median age 61 years) and is more common in male (male/female =2:1) [3].

T-PLL characterized by several clinical features like splenomegaly (75% of patients), generalized lymphadenopathy (50%), and skin infiltration (25%) with skin nodules, maculopapular rash, or more rarely erythroderma. Also serous effusions, particularly pleural effusions (15%) are regularly seen [4, 5]. The majority of T-PLL patients present with severely increased lymphocyte count (>100,000/μl). Anemia and thrombocytopenia are present in half of the patients, and lactate dehydrogenase (LDH) levels are usually elevated [6]. The clinical course of the disease is usually aggressive, with poor or no response to therapy [7].

The immunophenotype of T-PLL cells resembles that of mature post-thymic T-cells with expression of CD2, CD3, and CD7. The T-cell receptor (TCR) beta/gamma genes can be clonally rearranged [1]. The most frequent chromosomal abnormalities in T-PLL include 14q11.2-aberrations, chromosome 8 rearrangements and/or 11q abnormalities, the latter leading to the deletion of the ataxia-telangiectasia mutated (*ATM*) and mixed lineage leukemia (*MLL*) genes, as well as abnormalities e.g. in 5q, 6q, 9p, 12p, and 13q [8–11].

Here we report an untreated childhood T-PLL case with do novo del(9)(p13) as sole abnormality, which leads to monoallely of tumor suppressor gene *CDKN2A* (cyclin-dependent kinase inhibitor 2A).

## Material and methods

### Case report

A 16 year old male patient without significant personal or familial medical chronically presented with a 1 month history of dyspnea, fatigue, loss of weight, fever and pleural effusion in right lung. Pleural fluid examination revealed an elevated white blood cell (WBC) count of 218.4 × 10^9^/l (70% were lymphocytes and 15% were blasts), and LDH level of 3,486 U/l (normal up to 480 U/l). On physical examination enlarged liver was suggested whereas, CT scan and echography of the abdomen revealed normal liver size; several skin nodules (1–2 cm) in different locations such as neck, mandible and armpit were detected (data not shown). Routine peripheral blood test showed WBC count of 70.6 × 10^9^/l (74.6% were lymphocytes). The red blood cell (RBC) count was 4.52 × 10^6^/mm^3^ with a hemoglobin level of 11.8 g/dl and platelets count of 0.277 × 10^9^/l. Biochemistry analyses revealed LDH level of 1,377 U/l; alanine aminotransferase (ALT) level was 142 U/l (normal up to 40U/l); aspartate aminotrasferase (AST) level 51 U/l (normal up to 40U/l); total serum protein 6 g/dl (normal 6.4-8.3 g/dl); serum albumin 4 g/dl (normal 3.5-5.2 g/dl); and serum calcium value 9.7 mg/dl (normal 8.5-10.5 mg/dl). Bone marrow smear showed approximately 95% of cells were blasts. Unfortunately the patient died two months after diagnosis from the disease due to respiratory arrest.

### Cytogenetic analysis

Cytogenetic analysis using GTG-banding was performed according to standard procedures [12]. A minimum of 20 metaphase cells derived from unstimulated bone marrow culture were analyzed. Karyotypes were described according to the International System for Human Cytogenetic Nomenclature (ISCN 2009).

### Molecular cytogenetics

FISH using a whole chromosome painting (WCP) probe for chromosome 9 (MetaSystems/Germany) and a locus specific probe for *CDKN2A* gene (LSI p16 in 9p21) with a probe for centromere 9 (Abbott Molecular/Vysis, Abbott Park, IL, USA) were applied according to manufacturer’s instructions [12]. Also a multicolor banding probe (MCB) sets based on microdissection derived region-specific libraries for chromosome 9 was applied as previously described [13]. A minimum of 20 metaphase spreads were analyzed, using a fluorescence microscope (AxioImager.Z1 mot, Carl Zeiss Ltd., Hertfordshir, UK) equipped with appropriate filter sets to discriminate between a maximum of five fluorochromes plus the counterstain DAPI (4',6- diamino-2-phenylindole). Image capture and processing were performed using an ISIS imaging system (MetaSystems).

### Flow cytometry

Flow cytometry of leukemic blasts was performed using a general panel of fluorescent antibodies against the following antigens typical for different cell lineages and cell types: CD1a, CD2, CD3, CD4, CD5, CD8, CD10, CD11b, CD11c, CD13, CD14, CD15, CD16, CD19, CD20, CD22, CD23, CD32, CD33, CD34, CD38, CD41a, CD45, CD56, CD57, CD64, CD103, CD117, CD123, CD209, CD235a and CD243; in addition antibodies against Kappa and Lambda light Chains, sIgD, sIgM, and HLADr were applied (BD Biosciences). Four-color immunophenotyping on peripheral blood specimen was performed. Samples were stained and analyzed on a BD FACSCalibur™ flow cytometer according to BD Biosciences manuals and products insert sheets. Autofluorescence, viability, and isotype controls were included. Flow cytometric data acquisition and analysis were conducted by BD Cellquest™ Pro software.

## Results

**Figure 1:**
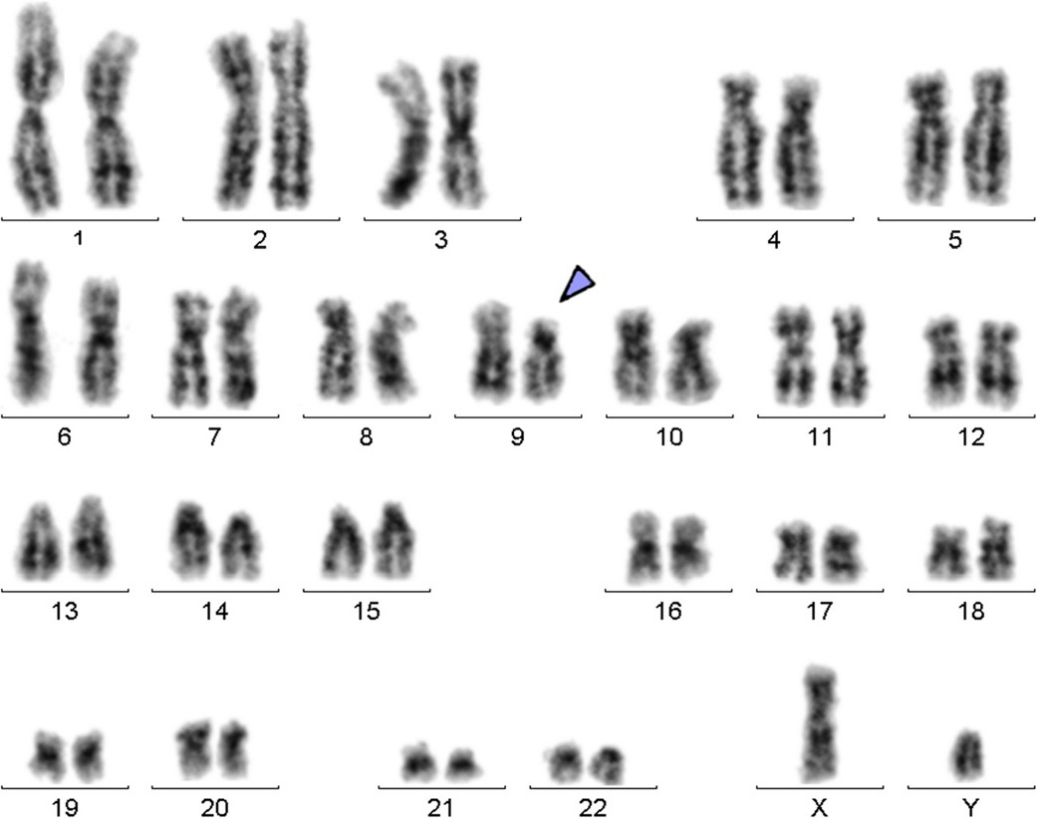
**GTG-banding revealed a deletion of the short arm of a derivative chromosome 9 del(9)(p?).** A derivative chromosome is marker by arrowhead.

**Figure 2:**
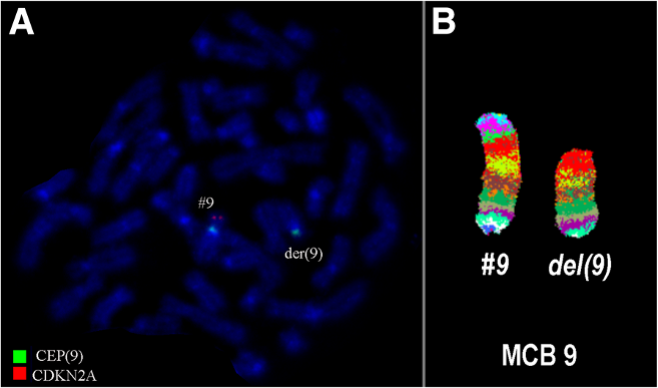
**Karyotype and chromosomal aberrations were confirmed using molecular cytogenetic approaches. (A)** The deletion of *CDKN2A* was identified on the der(9). **(B)** The application of MCB 9 characterized the del(9)(p13) comprehensively. Abbreviations: # = chromosome; der = derivative chromosome.

**Figure 3:**
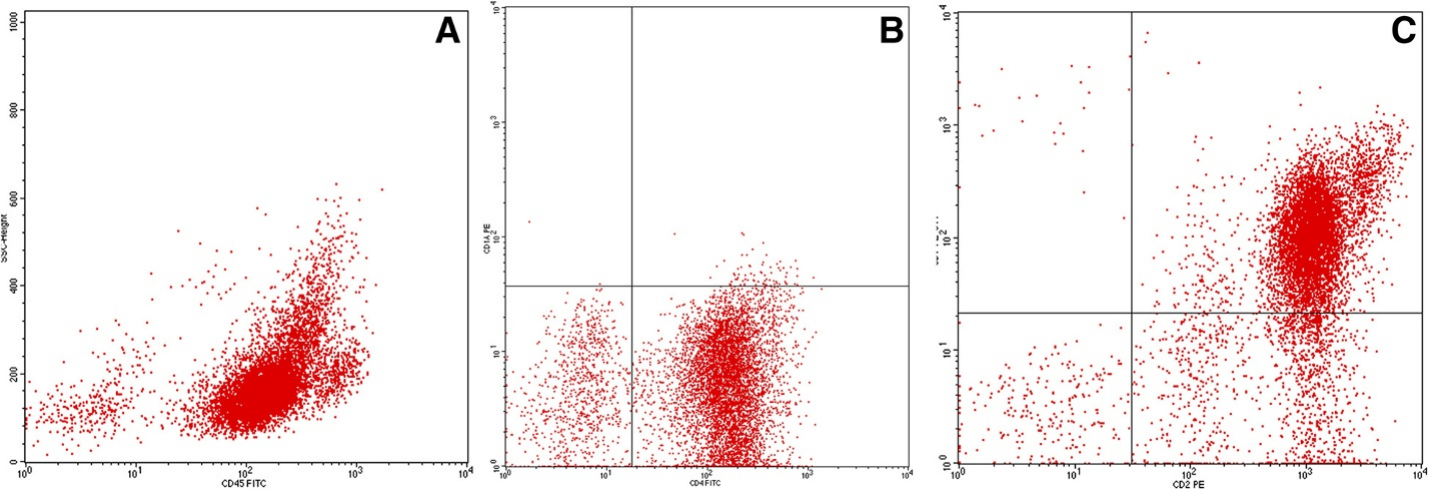
**Flow cytometric dot plots showing the abnormal population of T-cells. (A)** dim CD45 expression. **(B)** CD4 expression. **(C)** CD2 and CD7 coexpression.

Karyotyping was performed before initiation of any treatment and GTG banding revealed a karyotype of 46,XY,del(9)(p?) (20) (Figure 1). Dual color FISH was performed to confirm presence of the aberration. A probe specific for *CDKN2A* confirmed that the deletion encompassed subband 9p21 (Figure 2A). Finally, MCB9 probe set (Figure 2B) revealed the following karyotype: 46,XY,del(9)(p13) (20).Flow cytometric analysis of bone marrow specimen characterized this case as a T-PLL (Figures 3A-C). The abnormal cell population (97% of tested cells) was positive for CD45, CD2, CD4, CD7, and expressed CD5 heterogeneously and at low levels. Also the population was negative for TdT, CD1a, CD3, CD8, CD34, and HLA-DR.

## Discussion

T-PLL primarily affects older adults (median age at presentation, 61 years) with male predominance [3] and is considered as an aggressive leukemia with poor or no response to therapy [7].

T-cell CLL is now reclassified as T-PLL according to the WHO/revised form of European-American Classification of lymphoid neoplasm classification for lymphoid malignancies because of aggressive clinical behavior [14]. T-PLL represents approximately 2% of all mature lymphocytic leukemia in adults and 3% of T-cell malignancies overall [1, 15].

Concerning the immunophenotype of the present case, it fits into the known picture of T-PLL. Immunphenotype of T-prolymphocytes is consistent with that of a mature post-thymic T-cell. As such it was negative for CD1a and TdT, but positive for pan T-cell markers, such as CD2, CD3, CD5, and CD7. CD7 is expressed with stronger intensity than seen in normal T-cells and other mature T-cell malignancies, but at levels comparable to those seen in T-acute lymphocytic leukemia (T-ALL) [16]. Most of the cases (65%) have CD4+/CD8- cells, 21% of cases are CD4+/CD8+, and 13% of cases are CD4-/CD8+ [17]. Surface expression of CD3 and TCR-α/ß may not be detected in up to 20% of cases, but their expression is always seen in the cytoplasm [16].

As already mentioned T-PLL is a rare entity; here we describe a childhood T-PLL case with de novo del(9)(p13) leading to monoallelic deletion of the tumor suppressor gene *CDKN2A*. To the best of our knowledge, the present case is unique in two ways: it is the only one ever seen with this kind of aberration in a childhood case, and it is the only one with a del(9)(p13) as the sole aberration (for comparison see [18]).

The most frequent chromosomal abnormalities in T-PLL include 14q11.2-aberrations involving TCR-region, and 11q-rearrangements leading to deletions of *ATM* or *MLL* genes. Other yet reported abnormalities involve various chromosomal regions like 5, 6, 8, 9p, 7q, 12p, 13q, 14q, 16q, 17, 21 and 22 [8–11, 19, 20].

T-PLL occurs in enhanced frequencies in ataxia telangiectasia (AT) families [21]. This observation supports possible association between *ATM* mutations and T-PLL. Accordingly Friedenson [22] described ATM mutations in 44-66% of T-PLL patients.

*CDKN2A* and *CDKN2B* are tumor suppressor genes located in 9p21. They belong to the family of inhibitors of cyclin-dependent kinases. *CDKN2A* gene encodes for the two proteins p14^ARF^ and p16^INK4a^, and the *CDKN2B* gene for p15 ^INK4b^ protein; all three are key regulators of G1 phase cell-cycle arrest and senescence [23]. It has recently been observed that these genes are inactivated in a wide range of human cancers by epigenetic mechanisms [23, 24],

Loss of heterozygosity of chromosome arm 9p, including the *CDKN2A* locus, is one of the most frequent genetic events in childhood ALL, suggesting inactivation of the second allele or, possibly, haploinsufficiency [25]. Haploinsufficiency of a tumor suppressor gene (e.g. *CDKN2A*), has been shown to be adequate to promote tumor progression [26]. Homozygous deletion of *CDKN2A* has been suggested as the dominant mechanism of its inactivation in leukemogenesis [27].

FOXA1 is a key transcription (TF) factor for *CDKN2A* expression [24, 28] and a member of fork head gene family. TFs have remarkable pioneering activities to open chromatin for its subsequent cooperation with DNA-interacting proteins, especially important during embryogenesis and organ development [29, 30].

Our case fulfills the clinical, cytogenetic, molecular cytogenetic and flow cytometry criteria for a T-PLL case. It is important to recognize T-PLL because it has a more aggressive clinical course than mature T-cell leukemia. As del(9)(p13) was the only cytogenetic aberration in this aggressive childhood T-PLL case alterations of *CDKN2A* gene may be an early or even the primary event in T-PLL formation.

### Consent

Written informed consent was obtained from the patient for publication of this case report and accompanying images.

## References

[1] Maljaei SH, Brito-Babapulle V, Hiorns LR, Catovsky D (1998). Abnormalities of chromosomes 8, 11, 14, and X in T-prolymphocytic leukemia studied by fluorescence in situ hybridization. Cancer Genet Cytogenet.

[2] Oliveira F, Tone LG, Simones BP, Rego EM, Marinato AF, Jacomo RH, Falcao RP (2008). Translocations t(X;14)(q28;q11) and t(Y;14)(q12;q11) in T-cell prolymphocytic leukemia. Int J Lab Hematol.

[3] Dearden C (2012). How I treat prolymphocytic leukemia. Blood.

[4] Matutes E, Brito-Babapulle V, Yullie MR, Catovsky D, Cheson BD (2001). Prolymphocytic leukemia of B- and T-cell types. Chronic Lymphoid Leukemias.

[5] Catovsky D, Ralfkiaer E, Muller-Hermelink HK, Jaffe ES, Harris NL, Stein H, Vardiman JW (2001). T-cell prolymphocytic leukaemia. World Health Organization Classification of Tumours: Tumours of Haematopoietic and Lymphoid Tissues.

[6] Pawson R, Schulz TF, Matutes E, Catovsky D (1997). The human T-cell lymphotropic viruses type 1/11 are not involved in T Prolymphocytic leukaemia and large granular lymphocytic leukaemia. Leukemia.

[7] Brito-Bapapelle V, Pomfret M, Matutes E, Catovsky D (1987). Cytogenetic studies on prolymphocytic leukemia. II. T-cell pro-lymphocytic leukemia. Blood.

[8] Durig J, Bug S, Klein-Hitpass L, Boes T, Jons T, Subero-Martin JI, Harder L, Baudis M, Duhrsen U, Siebert R (2007). Combined single nucleotide polymorphism-based genomics mapping and global gene expression profiling identifies novel chromosomal imbalances, mechanisms and candidate genes important in the pathogenesis of T-Cell prolymphocytic leukemia with inv(14)(q11q32). Leukemia.

[9] Nowak D, Stern MH, Kawamata N, Akagi T, Dyer M, Hofmann WK, Ogawa S (2009). Molecular allelokaryotyping of T-cell prolymphocytic leukemia cells with high density single nucleotide polymorphism arrays identifies novel common genomic lesions and acquired uniparental disomy. Haematologica.

[10] Das D, Pathan S, Joneja M, Al-Musawi F, John B, Mirza K (2013). T-Cell Prolymphocytic Leukemia (T-PLL) with overlapping cytomorphological features with T-CLL and T-ALL: a case initially diagnosed by fineneedle aspiration cytology and lmmunocytochemistry. Diagn Cytopathol.

[11] Soulier J, Pierron G, Vecchione D, Garand R, Brizard F, Sigaux F, Stern MH, Aurias A (2001). A complex pattern of recurrent chromosomal losses and gains in T-cell prolymphocytic leukemia. Genes Chromosomes Cancer.

[12] AL-achkar W, Wafa A, Nweder MS (2007). A complex translocation t(5;9;22) in Philadelphia cells involving the short arm of chromosome 5 in a case of chronic myelogenous leukemia. J Exp Clin Cancer Res.

[13] Liehr T, Heller A, Starke H, Rubtsov N, Trifonov V, Mrasek K, Weise A, Kuechler A, Claussen U (2002). Microdissection based high resolution multicolor banding for all 24 human chromosomes. Int J Mol Med.

[14] Costa D, Queralt R, Aymerich M, Carrio A, Rozman M, Vallespi T, Colomer D, Nomdedeu B, Montserrat E, Campo E (2003). High levels of chromosomal imbalances in typical and small-cell variants of T-Cell prolymphocytic leukemia. Cancer Genet Cytogenet.

[15] Harris NL, Jaffe ES, Stein H, Banks PM, Chan JK, Cleary ML, Delsol G, De Wolf-Peeters C, Falini B, Gatter KC, Grogan TM, Isaacson PG, Knowles DM, Mason DY, Muller-Hermelink HK, Pileri SA, Piris MA, Ralfkiaer E, Warnke RA (1994). A revised European-American classification of lymphoid neoplasms: a proposal from the international lymphoma study group. Blood.

[16] Bartlett NL, Longo DL (1999). T-small lymphocyte disorders. Semin Hematol.

[17] Ginaldi L, Matutes E, Farahat N, De Martinis M, Morilla R, Catovsky D (1996). Differential expression of CD3 and CD7 in T-cell malignancies: a quantitative study by flow cytometry. Br J Haematol.

[18] Matutes E, Brito-Babapulle V, Swansbury J, Ellis J, Morilla R, Dearden C, Sempere A, Catovsky D (1991). Clinical and laboratory features of 78 cases of T-prolymphocytic leukemia. Blood.

[19] Mitelman F, Johansson B, Mertens F (2014). Mitelman Database of Chromosome Aberrations in Cancer.

[20] Salomon-Nguyen F, Brizard F, Le Coniat M, Radford I, Berger R, Brizard A (1998). Abnormalities of the short arm of chromosome 12 in T cell prolymphocytic leukaemia. Leukemia.

[21] Stankovic T, Kidd A, Sutcliffe A, McGuire G, Robinson P, Weber P, Bedenham T, Bradwell A, Easton D, Lennox G, Haites N, Byrd P, Taylor A (1998). ATM mutations and pheotypes in ataxia-telangiectasia families in the British isles: expression of mutant ATM and the risk of leukemia, lymphoma and breast cancer. Am J Hum Genet.

[22] Friedenson B (2007). The BRCA1/2 pathway prevents hematologic cancers in addition to breast and ovarian cancers. BMC Cancer.

[23] Collado M, Blasco MA, Serrano M (2007). Cellular senescence in cancer and aging. Cell.

[24] Zhang Y, Tong T (2014). FOXA1 antagonizes EZH2-mediated CDKN2A repression in carcinogenesis. Biochem Biophys Res Commun.

[25] Irving JA, Bloodworth L, Bown NP, Case MC, Hogarth LA, Hall AG (2005). Loss of heterozygosity in childhood acute lymphoblastic leukemia detected by genome-wide microarray single nucleotide polymorphism analysis. Cancer Res.

[26] Chapman EJ, Harnden P, Chambers P, Johnston C, Knowles MA (2005). Comprehensive analysis of CDKN2A status in microdissected urothelial cell carcinoma reveals potential haploinsufficiency, a high frequency of homozygous co-deletion and associations with clinical phenotype. Clin Cancer Res.

[27] Ohnishi H, Kawamura M, Ida K, Sheng XM, Hanada R, Nobori T, Yamamori S, Hayashi Y (1995). Homozygous deletions of p16/MTS1 gene are frequent but mutations are infrequent in childhood T-cell acute lymphoblastic leukemia. Blood.

[28] Li Q, Zhang Y, Fu J, Han L, Xue L, Lv C, Wang P, Li G, Tong T (2013). FOXA1 mediates p16(INK4a) activation during cellular senescence. EMBO J.

[29] Jozwik KM, Carroll JS (2012). Pioneer factors in hormone-dependent cancers. Nat Rev Cancer.

[30] Katoh M, Igarashi M, Fukuda H, Nakagama H, Katoh M (2013). Cancer genetics andgenomics of human FOX family genes. Cancer Lett.

